# Regional and patient-level determinants of endoscopic utilization in rural healthcare: a multi-level analysis

**DOI:** 10.3389/fonc.2025.1596332

**Published:** 2025-06-12

**Authors:** Muye Ma, Pengfei Li, Zhengyang Lu, Nan Zhang, Suzhen Wang, Youhua Lu, Jinming Yu

**Affiliations:** ^1^ School of Public Health, Shandong Second Medical University, Weifang, Shandong, China; ^2^ Shandong Cancer Hospital and Institute, Shandong First Medical University and Shandong Academy of Medical Science, Jinan, Shandong, China

**Keywords:** rural residents, upper digestive tract cancer, precancerous lesion, endoscopic examination, influencing factors

## Abstract

**Objective:**

Despite numerous studies on endoscopic services in urban settings, tailored assessments in rural healthcare remain limited, creating a gap in our understanding of resource-constrained environments. To address this gap, this study innovatively applied the Andersen Behavioral Model to systematically quantify endoscopic examination uptake and identify both patient-level (e.g., occupation, health history) and region-level (e.g., infrastructural challenges, socioeconomic indicators) factors influencing service utilization in rural China.

**Methods:**

We employed a multi-level logistic regression model with random intercepts to account for intraregional correlation and fixed effects for individual predictors. A multi-stage stratified random sampling approach was employed across 6 prefectures, yielding a sample of 1118 patients. We initially used descriptive statistics to summarize basic sample characteristics. Univariate analysis was then conducted to identify potential factors associated with endoscopic examination utilization. To further quantify these associations, we applied single-level and multi-level logistic regression model to account for potential regional effects and provide more robust analysis.

**Results:**

Of the 1,118 surveyed patients, 62.3% underwent endoscopic examinations, and among these, 77.9% received services at county-level institutions. In single-level binary logistic regression, region, occupation, household size, history of gastritis/esophagitis, and lesion location emerged as significant predictors (*P* < 0.05). In the multi-level logistic regression model, region remained a key factor, with the western region exhibiting 0.661 times lower odds (95% CI: 0.392–1.115) and the central region revealing 1.398 times higher odds (95% CI: 1.006–1.943) of service utilization compared to the eastern region. Additionally, unemployed status was associated with a 20% increased likelihood, and smaller household size correlated with a 87% increase in screening uptake.

**Conclusions:**

Our findings underscore the importance of addressing regional disparities through targeted resource allocation and localized health education programs to improve endoscopic service uptake among rural populations. These insights can inform policy interventions aimed at early cancer detection and optimized healthcare delivery in resource-limited settings.

## 1 Introduction

Upper gastrointestinal cancers (UGC), including malignancies of the esophagus, cardia, and stomach, are among the most prevalent and deadly cancers worldwide (1, 2). While high-income countries have benefitted from comprehensive screening programs, many low- and middle-income regions still experience high mortality rates, highlighting global disparities and the urgency for localized solutions. China, for example, bears a disproportionate burden, accounting for approximately 50% of global UGC cases and deaths (3, 4). This burden is particularly pronounced in rural regions, where limited healthcare resources and infrastructure deficits further exacerbate challenges in early detection and treatment (5). Therefore, promoting early detection and treatment, especially among high-risk populations, is crucial for reducing UGC mortality and improving patient outcomes.

Endoscopic examination, combined with pathological biopsy, is the gold standard for diagnosing UGC (6, 7) and plays an irreplaceable role in detecting cancerous and precancerous lesions. Recent advancements in endoscopic technology, including high-definition electronic endoscopy (8) and artificial intelligence (AI)-assisted diagnosis (2, 9), have significantly improved the safety and accuracy of the procedure (3). In addition to technological advancements, patient experience and subjective perception have become crucial dimensions in evaluating endoscopic screening quality. For instance, research based on the Service Quality Grid (SQG) provides an objective measure of endoscopic service quality, offering empirical support for optimizing screening procedures (10). Although previous studies (eg., (10, 11)) have examined patient satisfaction and service quality, they often overlook the interplay between socioeconomic determinants and screening behavior in rural settings. Surveys and interviews have been used to examine patient satisfaction across different screening protocols and service models. One study showed that overall satisfaction with UGC screening was highest in general satisfaction and lowest regarding convenience and accessibility. Key predictors included demographic and health factors, with residence and health self-assessment being most influential (11). Despite the rapid evolution of endoscopic diagnostic technology and the increased uptake of screening in urban settings, a significant research gap remains in understanding and addressing the multifaceted barriers, such as cultural, economic, and awareness-related factors, that uniquely affect rural populations.

To systematically analyze these multi-level determinants of endoscopic screening utilization, this study draws upon Andersen’s Behavioral Model of Health Services Use as a guiding theoretical framework. Andersen’s model posits that healthcare service utilization is shaped by three categories of factors: predisposing characteristics (such as age, gender, and education), enabling resources (including income, insurance, and access to health facilities), and need factors (such as perceived or evaluated health status) (12, 13). This model provides a structured approach to examining how both individual-level and contextual factors jointly influence rural residents’ decisions and abilities to undergo screening. Applying Andersen’s Behavioral Model allows for a comprehensive assessment of the demographic, socioeconomic, and health-related determinants of endoscopic screening in rural China, and supports the development of targeted interventions to address observed disparities.

In alignment with the model’s emphasis on perceived needs and enabling resources, several studies have investigated how participation in endoscopic screening is associated with improvements in health-related quality of life (HRQoL). While endoscopic procedures may cause temporary discomfort (14), the benefits of early diagnosis and timely intervention significantly improve survival rates and overall QoL. Compliance analysis reveals that factors such as cultural background, economic conditions (15), health literacy, and access to information influence participation in screening programs. These findings highlight the importance of considering socioeconomic factors when promoting UGC screening and developing targeted health promotion strategies to improve accessibility and acceptance.

Despite the growing application of endoscopic screening in clinical practice, significant disparities in accessibility persist across different regions and populations in China. In contrast to urban areas with abundant medical resources, rural patients face numerous challenges, including geographical remoteness, limited healthcare infrastructure, insufficient awareness, and financial constraints, resulting in lower utilization rates of endoscopic screening (16, 17). However, a clear understanding of the determinants behind the low endoscopic screening uptake among rural populations remains lacking, which hinders the design of effective policy interventions. This disparity not only delays early cancer detection but also exacerbates the overall disease burden, leading to poorer prognosis among rural patients. Therefore, conducting a systematic analysis of endoscopic service utilization among rural UGC and precancerous lesion patients and identifying influencing factors are crucial for optimizing medical resource allocation, increasing early detection rates, and formulating targeted policies.

This study aims to analyze the current utilization of endoscopic screening services among rural patients with UGC and precancerous lesions in China, explore the factors influencing their screening behavior, and propose strategies for optimizing screening programs. To accomplish the objectives, we conducted a cross-sectional study using a multistage stratified sampling design in rural healthcare settings. To address both the hierarchical structure of the data and missing observations, multi-level logistic regression and multiple imputation techniques were applied. Interpreting the findings within the Andersen’s Behavioral Model framework enhances our understanding of how multi-level determinants interact to influence endoscopic screening utilization in rural China. These findings provide the basis and reference for promoting rural patients to seek timely medical treatment, optimizing related management strategies and improving the health of the population.

## 2 Methods

### 2.1 Study design and participants

This cross-sectional study was conducted in 2019 to investigate factors influencing endoscopic service utilization among rural patients diagnosed with UGC and precancerous lesions in Shandong Province, China. A multi-stage stratified random sampling method was employed to select a representative sample from different regions within the province. To capture structural disparities, Shandong Province was stratified based on a combination of geographic location (eastern, central, and western) and economic development levels. This dual-criteria approach was intended not to reflect strict geographic or administrative boundaries, but rather to facilitate region-level comparisons in the context of multi-level modeling. Such stratification has been widely adopted in public health research aimed at assessing disparities in healthcare utilization and service distribution across macro-regions in China (18). A total of six prefecture-level cities were selected: Jinan and Weifang from the eastern region, Jining and Tai’an from the central region, and Liaocheng and Binzhou from the western region. Although Jinan is often classified as a central city geographically, it was grouped into the eastern region in this study due to its higher development level and rural coverage characteristics. This decision was made to create more meaningful contrast with rural areas in the central and western regions, rather than to strictly adhere to administrative geography. Within each selected city, corresponding counties, districts, or rural areas were randomly chosen to conduct on-site surveys. This approach allowed for a wide coverage of rural communities across the province.

The study targeted rural patients who met specific inclusion criteria. Participants were individuals aged 18 years or older with a confirmed diagnosis of UGC, including esophageal or gastric cancer, or precancerous lesions. They resided in rural areas within the selected counties or districts and possessed the cognitive ability to understand the questionnaire and provide informed consent. Additionally, participants demonstrated a willingness to engage in the study by agreeing to complete the survey. Patients were identified through collaboration with local hospitals, cancer registries, and community health centers, with healthcare providers play a crucial role in informing eligible individuals about the study during routine consultations or follow-up visits.

Out of an estimated 1,500 potential participants identified via local registries, 1,118 met the inclusion criteria after exclusion of those with incomplete records or inability to consent. This sizable and diverse sample allowed for robust analysis of factors influencing endoscopic service utilization among rural populations in Shandong Province.

In line with the study’s aim to systematically assess multi-level determinants of service utilization, the subsequent analysis was guided by Andersen’s Behavioral Model of Health Services Use. Specifically, variables collected on participant demographics, socioeconomic status, health conditions, and related factors were grouped into predisposing, enabling, and need factors according to Andersen’s framework. This approach facilitated a theory-driven examination of the influence of individual and contextual factors on endoscopic screening utilization in rural communities.

### 2.2 Data collection and variable classification based on Andersen’s behavioral model

Data was collected using a structured questionnaire developed by the research team, which was administered through interviews. The questionnaire consisted of seven sections, designed to collect detailed information on various factors influencing endoscopic service utilization and cancer prevention behaviors among rural patients. The sections are as follows: basic information, behavioral and living habits, clinical information, information related to health insurance, medical and non-medical cost collection, QoL status and cancer prevention needs. Interviewers underwent a standardized training session that included role-playing and calibration exercises to ensure uniform administration of the questionnaire.

To ensure that the analytical framework aligned with established health behavior theory, all variables collected through the questionnaire were subsequently classified according to Andersen’s Behavioral Model of Health Services Use, which categorizes determinants of healthcare utilization into three domains:

Predisposing factors refer to inherent individual characteristics that influence the propensity to seek care. In this study, these included demographic and social structure variables such as Gender, Age group, Education level, Marital status, Occupation, Household size, Behavioral and lifestyle habits (i.e., smoking status, alcohol drinking).

Enabling factors represent the means and resources that facilitate or hinder access to healthcare services. These included: Region (Western, Central, Eastern), Family annual income and Insurance status, all of which reflect structural conditions affecting healthcare access in rural settings.

Need factors capture the perceived or clinically evaluated health status that directly prompts service utilization. These covered: History of gastritis/esophagitis, Use of medications for gastritis/esophagitis, Lesion location (esophagus, cardia, stomach), Use of Medications, and comprehensive assessments of QoL.

Prior to the main survey, the questionnaire underwent pilot testing to assess its clarity, relevance, and cultural appropriateness. The pilot test involved 30 patients reflecting diverse demographic backgrounds, which led to modifications in the wording of 5 key questions related to medical cost perception. Additionally, completed questionnaires were reviewed on-site to verify completeness and resolve any discrepancies immediately. This on-the-spot data verification minimized missing data and improved the overall reliability of the information collected.

### 2.3 Ethical considerations

Informed consent was obtained from all participants prior to survey administration. Participants received comprehensive information about the purpose of the study, the procedures involved, their rights as participants, and the voluntary nature of their involvement. They were explicitly informed of their right to withdraw from study at any time without any consequences. Consent was documented in writing to ensure transparency and adherence to ethical guidelines. Confidentiality and anonymity were rigorously maintained throughout the study. Personal identifiers were not collected; instead, questionnaires were coded to protect participant identities. All data were securely stored in a password-protected database with access limited to authorized members of the research team. The study was conducted in accordance with the ethical standards of the Declaration of Helsinki and its subsequent amendments or comparable ethical standards. Ethical approval for this study was granted by the Ethics Committee of Shandong Institute of Cancer Prevention and Control, with the approval ID: SDTHEC201909001.

### 2.4 Statistical analysis

This study was conducted by means of an on-site survey, with questionnaires administered by trained investigators under strict quality control. Data was entered and analyzed using SPSS 26.0. The baseline characteristics of the study participants and the utilization of endoscopy services were descriptively analyzed using frequency distributions (i.e., composition ratios).

To address missing data beyond on-site verification, we implemented a multiple imputation strategy. Specifically, we applied a chained equations approach where continuous variables were imputed via linear regression and categorical variables via logistic regression. This imputation process was repeated for five iterations, producing five complete datasets. By retaining partially observed data, this method reduces the bias associated with a complete-case analysis, and Rubin’s rules were used to combine the results, yielding final estimates that account for the uncertainty from the imputation process.

For inferential analysis, statistically significant variables from univariate analyses (chi-square test and Fisher’s exact test for unordered categorical variables, and the rank-sum test for ordered categorical data) were considered as candidates for multivariable modeling, along with variables that were deemed clinically important according to the Andersen framework. To explore determinants of endoscopy service utilization, both single-level and multi-level logistic regression models were employed. Recognizing the potential for a hierarchical data structure (e.g., patients tested within regions or health facilities), multi-level logistic regression models were applied to account for clustering effects. The multi-level structure was defined based on geographic or facility-based groupings of patients. In all regression models, variable inclusion was guided by statistical significance from univariate screening as well as clinical relevance.

Model assumptions were thoroughly evaluated, including assessment of multicollinearity (using variance inflation factors) and the linearity of continuous predictors. Goodness-of-fit was assessed with the Hosmer-Lemeshow test and residual analysis. A two-sided test level of α = 0.05 was used, with *P* < 0.05 considered to indicate statistically significant findings.

## 3 Results

### 3.1 Characteristics of patients

This study surveyed a total of 1,118 patients with upper gastrointestinal cancer or precancerous lesions in rural areas of Shandong Province, China. The results indicated that most patients originated from the eastern (48.0%) and central (40.7%) regions. Of the total, 780 patients (69.8%) were male and 338 (30.2%) were female. A significant proportion of patients were aged 60 years or older (71.5%), with the vast majority being married (90.4%) and having an education level of junior high school or below (85.6%). Regarding occupation, 83.3% were farmers or migrant workers. In terms of household characteristics, 55.2% of patients lived in households with two permanent residents, and 94.2% had a household annual income of less than 50,000 yuan. Additionally, 97.6% of the patients participated in urban and rural resident medical insurance, reflecting a high rate of insurance coverage.

Regarding medical history, 39.2% of patients reported a history of gastritis or esophagitis, and 26% had taken medications related to these conditions. As for lifestyle habits, 53.6% of patients were current or former smokers, while 55.6% were had a history of alcohol consumption. The distribution of lesion sites showed that the cancer or precancerous lesions were primarily located in the esophagus and stomach, accounting for 47.4% and 37.1%, respectively. Detailed information is provided in **Table 1**, grouped according to the Andersen Behavioral Model.

**Table 1:** Baseline characteristics of patients with upper gastrointestinal cancer or precancerous lesions (n = 1118).

Variables	n (%)	Variables	n (%)
Predisposing Factors			
Gender		Occupation	
Male	780(69.8)	Civil servant	19(1.7)
Female	338(30.2)	Worker	26(2.3)
Age group (y)		Employee	1(0.1)
<50	46(4.1)	Self-employed	23(2.1)
50–59	273(24.4)	Freelancer	11(1.0)
60–69	542(48.5)	Farmers/migrant workers	930(83.3)
70–79	233(20.8)	Unemployed	36(3.2)
≥80	24(2.2)	Retired	72(6.4)
Education level		Student	0(0.0)
Illiterate	181(16.2)	Smoking	
Primary school	357(31.9)	Never smokers	518(46.3)
Junior high school	419(37.5)	Current smokers	205(18.3)
High school or above	161(14.4)	Former smokers	395(35.3)
Marital Status		Alcohol drinking	
Unmarried	12(1.1)	Never drinker	496(44.4)
Married	1011(90.4)	Current drinker	255(22.8)
Divorced	4(0.4)	Former drinker	367(32.8)
Widowed	91(8.1)		
Household size			
1 person	89(8.0)		
2 people	617(55.2)		
3 or more people	412(36.8)		
Enabling Factors			
Region		Insurance status	
Western	126(11.3)	Urban employee basic medical insurance	71(6.4)
Central	455(40.7)	Urban and rural resident basic medical insurance	1020(0.5)
Eastern	537(48.0)	Commercial medical insurance	6(0.5)
Family annual income (CNY)		Public Medical Insurance	3(0.3)
<10,000	525(47.0)	Self-paid	8(0.7)
10,000–50,000	393(35.1)	Others	4(0.4)
50,000–10,000	135(12.1)	Unclear	6(0.5)
>10,000	65(5.8)		
Need Factors			
History of Gastritis/Esophagitis		Lesion location	
Yes	438(39.2)	Esophagus	530(47.4)
No	680(60.8)	Cardia	173(15.5)
Use of Medications for Gastritis/Esophagitis		Stomach	415(37.1)
Yes	291(26.0)		
No	827(74.0)		

### 3.2 Univariate analysis of factors influencing the utilization of endoscopy services

**Figure 1:**
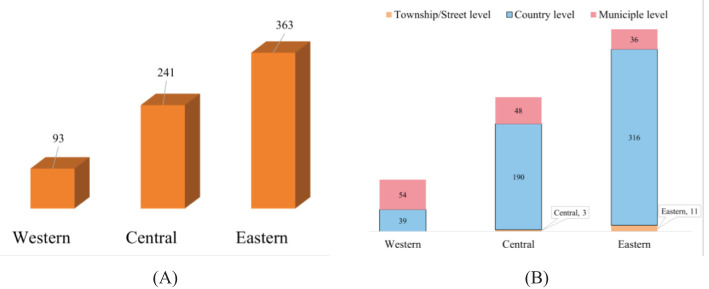
**(A)** Distribution of endoscopic service utilization across regions; **(B)** Distribution of healthcare institutions by administrative level.

The survey indicated that 62.3% of patients utilized endoscopic examination services, with the majority seeking care at county-level healthcare institutions. Notably, township- or street-level facilities (2.2%) were rarely chosen, highlighting potential disparities in accessibility or trust in lower-level facilities. The overall utilization patterns and specific locations for endoscopic examinations are detailed in **Figures 1A, B**.

To further explore these patterns, a univariate analysis was performed using chi-square and Fisher’s exact tests for categorical variables. This analysis revealed that several variables were significantly associated (*P* < 0.05) with the utilization of endoscopic examination services among rural patients with upper gastrointestinal cancer or precancerous lesions.

Among the predisposing factors, age, marital status, occupation, and the number of permanent household members showed statistically significant associations with endoscopy utilization (*P* < 0.05 for each). In contrast, other predisposing variables such as gender, educational attainment, and lifestyle factors, including smoking and alcohol consumption, did not demonstrate significant associations. Regarding enabling factors, region emerged as a significant determinant, with marked differences in utilization rates across geographic areas (*P* < 0.05), while neither household income nor insurance status were significantly related to service uptake. Analysis of need factors revealed that a history of gastritis or esophagitis, use of medications for these conditions, and the anatomical location of the cancer or precancerous lesion were each significantly associated with endoscopy utilization (all *P* < 0.05).

These findings offer a comprehensive view of the factors influencing endoscopic service utilization and underscore the importance of addressing both demographic and clinical variables in optimizing healthcare delivery in rural settings. Detailed results for all variables are presented in **Table 2**.

**Table 2:** Univariate analysis of factors associated with endoscopic examination use.

Variables	*χ^2^*	*P*
Predisposing Factors		
Gender	0.194	0.659
Age group	–	<0.05^*^
Education level	1.516	0.679
Marital Status	8.599	0.027^*^
Occupation	140.057	<0.001^***^
Household size	20.880	<0.001^***^
Smoking status	0.145	0.930
Alcohol drinking	0.975	0.614
Enabling Factors		
Region	30.411	<0.001^***^
Family annual income (CNY)	–	>0.05
Insurance status	–	>0.05
Need Factors		
History of Gastritis/Esophagitis	29.476	<0.001^***^
Use of Medications for Gastritis/Esophagitis	–	<0.001^***^
Lesion location	33.528	<0.001^***^

^*^ *P* < 0.05, ^**^ *P* < 0.01, ^***^ *P* < 0.001; Fisher’s exact test was used for variables with expected frequencies < 5; For variables with missing data handled via multiple imputation, exact *P*-values could not be calculated. Accordingly, significance was assessed using the threshold of *P* < 0.05.

### 3.3 Binary logistic regression analysis

Taking the utilization of endoscopic examination services as the dependent variable, factors with statistical significance from the univariate analysis were included as independent variables in a binary logistic regression model. A forward stepwise regression strategy was applied to select independent variables, and dummy variables were created for multi-level categorical variables using the last level as the reference. Prior to final model implementation, multicollinearity was assessed. The results showed that all tolerance values exceeded 0.1, and all variance inflation factor (VIF) values were below 10, suggesting that was not a significant concern in this study.

Multiple interpolations were conducted by the chained equations approach to address missing data. Notably, the age variable reached significance (*P* < 0.05) in three of the imputed datasets, while it failed to reach significance in the remaining datasets. Further sensitivity analyses showed that the odds ratio (OR) and statistical significance of the other variables remained consistent regardless of whether the age variable was included or not. This suggests that age has a negligible overall effect on the model and therefore the age variable was not included in the final model to improve the robustness and interpretability of the model.

Model goodness-of-fit was evaluated using the Hosmer-Lemeshow test (χ^2^ = 3.866, *P* = 0.869, with significance level *α* = 0.05). The failure to reject the null hypothesis suggests no significant difference between observed and predicted values, indicating a good model fit.

The final regression model (**Table 3**) identified several significant predictors, which can be interpreted within the framework of Andersen’s Behavioral Model. Among enabling factors, region was a significant factor, with patients from the central region more likely to utilize endoscopic services than those from the eastern region (OR = 1.422, *P* = 0.015). Regarding predisposing factors, occupation type was significant. Unemployed individuals had significantly lower odds of utilizing endoscopic services compared to retirees (OR = 0.214, *P* = 0.003), suggesting that financial constraints or reduced healthcare access may contribute to this difference. In addition, the number of permanent household members also influenced service utilization. Patients from households with two members were less likely to utilize endoscopic services compared to those from single-member households (OR = 0.214, *P* = 0.015). For need-related variables, the history of gastritis or esophagitis was positively associated with endoscopic services utilization (OR = 0.482, *P* < 0.001). Patients with such a medical history might be more vigilant about their gastrointestinal health and therefore more likely to undergo endoscopic examinations. Lastly, the location of cancer or precancerous lesions also influenced behaviors. Patients with esophageal lesions were less likely to utilize endoscopic services than those with cardia lesions (OR = 2.051, *P* < 0.001), suggesting that differences in symptom presentation or perceived severity may affect the decision to undergo endoscopic examination.

**Table 3:** Significant predictors of endoscopic examination utilization identified by binary logistic regression analysis.

Variables	β	P	OR	95%CI
Predisposing Factors				
Occupation		0.119		
Civil servant	-0.963	0.129	0.382	0.110–1.323
Worker	0.187	0.702	1.205	0.464–3.134
Employee	21.694	1.000		
Self-employed	-0.454	0.372	0.635	0.234–1.720
Freelancer	-21.079	0.999	0.000	0.000
Farmers/migrant workers	-0.273	0.293	0.761	0.457–1.266
Unemployed	-1.540	0.003^*^	0.214	0.077–0.598
Retired			1.000	
Household Size		<0.001^***^		
1 person	0.103	0.676	1.108	0.684–1.795
2 people	-0.609	<0.001^***^	0.544	0.413–0.716
3 or more people			1.000	
Enabling Factors				
Region		0.001^**^		
Western	-0.466	0.046^*^	0.627	0.397–0.992
Central	0.352	0.015^*^	1.422	1.070–1.890
Eastern			1.000	
Need Factor				
History of gastritis/esophagitis				
Yes	0.730	<0.001^***^	0.482	0.367–0.632
No			1.000	
Lesion location		<0.001^***^		
Esophagus	-0.718	<0.001^***^	2.051	1.522–2.764
Cardia	-0.287	0.162	1.333	0.891–1.993
Stomach			1.000	
Constant	-0.113	0.700	0.893	0.504–1.584

^*^ *P* < 0.05, ^**^ *P* < 0.01, ^***^ *P* < 0.001. The reference groups for categorical variables are explicitly defined: for Region, the eastern region is the reference; for Occupation, the retired group is used as the reference; for Household Size, households with 3 or more people serve as the reference; for History of Gastritis/Esophagitis, “No” is the reference; and for Lesion Location, the stomach is the reference.

### 3.4 Multi-level logistic regression analysis

This study utilized a multistage stratified sampling method, collecting data at two distinct levels. Level 1 represents individual patient data, capturing patient-specific characteristics and outcomes, while Level 2 comprises regional characteristics that may influence service utilization. Given this hierarchical structure, a multi-level logistic regression model was employed to appropriately account for clustering within regions and potential regional-level confounders. This approach ensured that both within-region and between-region variability were accurately captured, thereby providing more reliable estimates of the factors influencing the utilization of endoscopic examination services among rural patients with upper gastrointestinal cancer or precancerous lesions.

As illustrated in **Table 4**, the null model (without predictors) yielded a fixed intercept of β = -0.611 (*P* = 0.023), indicating that the log-odds of utilizing endoscopic examination services were negative and statistically different from zero. The estimated random intercept variance for Region was 0.197 (*P* = 0.362), suggesting that although some variability exists at the regional level, it is not statistically significant (*P* > 0.05).

**Table 4:** Null model for multi-level logistic regression analysis.

Effects	Estimates	SD	*Z*	*P*
Fixed Effects (Intercept)	-0.611	0.268	2.282	0.023
Random effect (Level 2: Region)	0.197	0.216	0.911	0.362

Given the lack of significant random variability at the regional level, Region was subsequently added as a fixed effect along with individual-level predictors. Treating Region as a fixed effect—rather than as a random effect—was justified by the minimal between-region variance, allowing a more precise estimation of regional differences in service utilization.

**Table 5** presents the results of the multi-level logistic regression analysis, which highlights several important factors influencing endoscopic service utilization. Notably, Region was found to significantly impact utilization, with the western region (OR = 0.661) showing lower odds and the central region (OR = 1.398) showing higher odds of utilizing endoscopic services compared to the eastern region, which served as the reference group. Occupation had limited significant effects on utilization (*P* = 0.294). The unemployed group showed a statistically significant negative association with endoscopic service utilization (OR = 0.273, *P* = 0.021), suggesting that unemployed individuals are significantly less likely to utilize endoscopic services. Household size was a significant factor, with individuals from smaller households (especially 2-person households) being less likely to utilize endoscopic services (*P* = 0.001, OR = 0.573). History of gastric/esophageal disease was a strong predictor, with those having a history of these conditions being significantly more likely to undergo endoscopy (*P* = 0.016). Lesion location had a significant effect on utilization, particularly for individuals with esophageal lesions, who were more likely to use endoscopic services (*P* < 0.001, OR = 2.167).

**Table 5:** Multi-level logistic regression analysis.

Variables	β	*P*	OR	95%CI
Predisposing Factors				
Occupation		0.294		
Civil servant	−0.661	0.328	0.516	0.137–1.945
Worker	0.252	0.653	1.286	0.428–3.864
Employee	23.373			
Self-employed	−0.596	0.340	0.551	0.162–1.878
Freelancer	−22.252	0.999		
Farmers/migrant workers	−0.251	0.390	0.778	0.438–1.381
Unemployed	−1.299	0.021^*^	0.273	0.090–0.823
Retired			1.000	
Household Size		0.004^**^		
1 person	−0.135	0.723	0.873	0.413–1.849
2 people	−0.557	0.001^**^	0.573	0.412–0.796
3 or more people			1.000	
Enabling Factors				
Region		0.013^*^		
Western	−0.414	0.120	0.661	0.392–1.115
Central	0.335	0.046^*^	1.398	1.006–1.943
Eastern			1.000	
Need Factor				
History of gastritis/esophagitis				
Yes	−0.524	0.016^*^	0.592	0.387–0.906
No			1.000	
Lesion location		<0.001^***^		
Esophagus	0.773	<0.001^***^	2.167	1.529–3.073
Cardia	0.393	0.097	1.481	0.931–2.355
Stomach			1.000	

^*^ *P* < 0.05, ^**^ *P* < 0.01, ^***^ *P* < 0.001. The reference groups for categorical variables are explicitly defined: for Region, the eastern region is the reference; for Occupation, the retired group is used as the reference; for Household Size, households with 3 or more people serve as the reference; for History of Gastritis/Esophagitis, “No” is the reference; and for Lesion Location, the stomach is the reference.

Both the multi-level and binary logistic regression analyses highlight the significant influence of regional and individual-level factors on the utilization of endoscopic examination services, with each method providing complementary insights. While the multi-level analysis allowed for the assessment of regional variability—even though the regional random effect was not significant, it confirmed that incorporating Region as a fixed effect was necessary. In contrast, the binary logistic regression model, by focusing solely on individual-level predictors, underscored similar factors. Moreover, it revealed that occupation, household size, history of gastritis/esophagitis, and lesion location significantly impacted service utilization.

Both approaches consistently identify key predictors as central to understanding the utilization of endoscopic services. The binary logistic model, with its simpler structure, allows for clear interpretation of these relationships, while the multi-level model accounts for the data’s hierarchical nature and provides insight into potential regional heterogeneities. Together, these analyses offer a comprehensive view of the factors influencing the use of endoscopic services among rural patients with upper gastrointestinal issues, allowing for more targeted public health interventions.

## 4 Discussion

This study identified several key factors influencing the utilization of endoscopic examination services among rural patients with UGC or precancerous lesions. Both the binary and multi-level logistic regression analyses highlighted the importance of regional differences, occupation, household size, history of gastritis/esophagitis, and lesion location in shaping patients’ likelihood of seeking endoscopic care. Notably, unemployed individuals and those from smaller households (*P* < 0.05) were less likely to utilize endoscopy services, whereas having a history of gastritis/esophagitis or esophageal lesions was associated with higher odds of service use. These findings reflect the combined influence of predisposing, enabling, and need factors, as theorized in Andersen’s Behavioral Model of Health Services Use and underscore the need for targeted interventions addressing socio-demographic and clinical characteristics to enhance endoscopic service uptake in rural settings.

Regional disparities (enabling factors) emerged as a key predictor in both binary and multi-level regression analyses. Among the 697 patients who underwent endoscopic screening, utilization rates varied markedly by region: 8.3% in the western region, 21.6% in the central region, and 32.5% in the eastern region. These discrepancies likely reflect variations in local healthcare infrastructure, the accessibility of county-level institutions, and regional public health policies. In the eastern region, for example, the presence of well-equipped hospitals and specialized endoscopy centers, along with a higher density of healthcare professionals, seems to contribute to the higher uptake of screening services. In contrast, the western region often struggles with limited resources, fewer tertiary care facilities, and longer travel distances for patients, which may hinder timely access to endoscopic services. Importantly, transportation barriers and longer travel times are critical determinants of endoscopic service utilization in rural areas. Many patients face significant distances and limited transport options, leading to increased indirect costs and reduced screening uptake (19). Our findings of lower utilization in less resourced regions align with previous studies and underscore the need to address transportation and access in efforts to reduce disparities in preventive care. It is important to note that the shift from random to fixed effects for Region enabled the detection of systematic regional differences, highlighting that while the overall variance across regions may appear modest in the random effects model, structured infrastructural differences—such as those witnessed in the eastern region’s healthcare provision—still drive utilization rates.

Furthermore, the role of county-level institutions cannot be underestimated. In regions where these institutions are well-supported, screening services are more consistently available, and patients benefit from streamlined referral processes. Conversely, in areas where such institutions are under-resourced, gaps in service provision and follow-up care might contribute to lower overall utilization rates. These infrastructural differences also interplay with regional public health policies: regions with proactive initiatives—such as subsidized screening programs, targeted public awareness campaigns, and enhanced insurance coverage—tend to exhibit greater uptake of preventive services. Similar patterns have been noted in other Chinese provinces and in developing countries, where unequal distribution of healthcare resources often leads to regional clustering of health service use (20, 21).

The markedly lower endoscopic utilization rates in the western region highlight the need for decentralizing endoscopic infrastructure to increase accessibility and reduce travel burdens for rural populations. Policymakers should prioritize investments in building and equipping endoscopy facilities at the county and township levels in under-resourced areas, particularly in the western region. Additionally, the observed lower utilization rates among larger households suggest that tailored health education and outreach interventions are warranted. Public health agencies should develop and implement targeted health education campaigns for larger households, addressing specific barriers such as lack of awareness about the importance of early screening. Strengthening both the physical infrastructure for endoscopic services and targeted education efforts will be crucial for reducing disparities and improving cancer screening uptake in rural China.

Previous research has recognized the importance of examining hierarchical clustering in chronic disease analysis (22, 23). For instance, studies have demonstrated that single-level logistic regression models may mask the effects of regional and institutional heterogeneity that are critical for understanding healthcare service utilization (24). A cross-sectional survey of 861 Jordanians aged 50–75 reported that only 17.2% underwent colorectal cancer screening, despite more than one-third reporting awareness of its necessity. Key barriers included “feeling well,” (i.e., believing screening is unnecessary due to lack of symptoms), lack of physician endorsement, and difficult healthcare access (25). Similarly, a US-based study examining county-level social vulnerability and screening rates for breast, cervical, and colorectal cancers showed that counties with a higher social vulnerability index (SVI) consistently exhibited lower odds of screening (26), even after adjusting for urban-rural status, uninsured rate, and primary care physician supply. Similar disparities have been reported internationally. A multi-country survey in eastern sub-Saharan Africa found extremely limited endoscopy capacity—only 0.12 endoscopists and 0.12 functioning gastroscopes per 100,000 people—with equipment shortages, lack of trained personnel, and high costs as key barriers (27). Likewise, a study from Pakistan showed that lower gastrointestinal malignancies were more common among patients from low socioeconomic backgrounds, reflecting delayed access to diagnostic services (28). These findings align with our results in rural China, reinforcing the global relevance of addressing infrastructure and social vulnerability in cancer screening.

In our study, the use of a multi-level logistic regression model to analyze screening utilization provided additional insights that align with these previous findings. Unlike conventional single-level models that may conceal important variations, our hierarchical approach captures both individual and area-level influences—thereby delineating more accurately the factors associated with disparate screening rates. Although the random effect of regions in the null model did not reach statistical significance (*P* = 0.362), the analysis clearly demonstrated that when region was incorporated as a fixed effect, significant variations in utilization between the western, central, and eastern regions emerged. Our application of a hierarchical model not only refines estimates of screening determinants but also offers a framework for analyzing similar preventive services in resource-limited settings—an approach that aligns with emerging trends in healthcare analytics. This observation is consistent with the work of previous investigators, reinforcing the notion that a more nuanced model capturing both individual and area-level factors is essential for accurately identifying barriers and facilitators to endoscopic service utilization.

Several limitations should be acknowledged. First, although multi-level logistic regression was appropriate for the hierarchical structure of the data, the random effect of region was not significant in some models, possibly due to limited sample size or unmeasured heterogeneity within regions. Second, the cross-sectional design restricts our findings to associations rather than causality; longitudinal studies are needed to clarify temporal relationships between predictors and endoscopic utilization. Third, despite including a range of variables guided by Andersen’s Behavioral Model, unmeasured factors such as detailed socioeconomic status, health literacy, cultural beliefs, and attitudes towards cancer screening may have introduced residual confounding. Fourth, the study did not account for potential variations in endoscopic service quality, such as equipment standards or operator expertise, which could influence both utilization patterns and patient willingness to undergo screening. Lastly, the broad geographic stratification into eastern, central, and western regions, while practical, may obscure important subregional differences in infrastructure and cultural practices. Our study did not directly assess cultural beliefs or attitudes toward cancer screening, which are known to significantly influence health-seeking behavior in rural China. Future research should employ larger and more granular samples, collect longitudinal data, incorporate quality metrics, and use mixed-methods approaches to more comprehensively understand the determinants of endoscopic service utilization.

In summary, the application of multi-level logistic regression analysis and Andersen’s Behavioral Model in our study provides more reasonable statistical support for understanding the determinants of endoscopic examination service utilization among patients in rural Shandong Province. By explicitly comparing our hierarchical modeling approach with traditional single-level methods, our study not only enhances the credibility of the findings but also establishes a methodological precedent for future investigations of preventive healthcare services in diverse and resource-constrained settings. This methodological approach offers valuable experience in addressing hierarchical clustering issues and has important implications for optimizing healthcare resource allocation and enhancing public health service delivery.

## Data Availability

The original contributions presented in the study are included in the article/supplementary material. Further inquiries can be directed to the corresponding author.

## References

[1] Yang H Wang JB Zhang JY Fan JH Qiao YL Taylor PR. Family history and risk of upper gastrointestinal cancer in the linxian general population. Front Oncol. (2021) 11:605106. doi: 10.3389/fonc.2021.605106 34123779 PMC8193945

[2] Huang XY Qin MH Fang MJ Wang ZP Hu CE Zhao TY. The application of artificial intelligence in upper gastrointestinal cancers. J Natl Cancer Center. (2024) 5:S266700542400125X. doi: 10.1016/j.jncc.2024.12.006 PMC12010392 40265096

[3] Zeng HM Sun KX Cao MM Zheng RS Sun XB Liu SZ. Initial results from a multi-center population-based cluster randomized trial of esophageal and gastric cancer screening in China. BMC Gastroenterol. (2020) 20:398. doi: 10.1186/s12876-020-01517-3 33228549 PMC7686770

[4] Chen R Liu Y Song GH Li BY Zhao DL Hua ZL. Effectiveness of one-time endoscopic screening programme in prevention of upper gastrointestinal cancer in China: a multicentre population-based cohort study. Gut. (2020) 70:gutjnl–2019-320200. doi: 10.1136/gutjnl-2019-320200 PMC7815635 32241902

[5] Wang H Liu Z Guo CH Liu MF He Y Tian HR. Health-seeking behavior and barriers to treatment of patients with upper gastrointestinal cancer detected by screening in rural China: real-world evidence from the ESECC trial. Lancet Regional Health - Western Pacific. (2021) 12:100181. doi: 10.1016/j.lanwpc.2021.100181 34527972 PMC8356128

[6] Miura Y Osawa H Sugano K. Recent progress of image-enhanced endoscopy for upper gastrointestinal neoplasia and associated lesions. Dig Dis. (2024) 42:186–98. doi: 10.1159/000535055 37952532

[7] Yoo JW Laszkowska M Mendelsohn RB. The role of screening and early detection in upper gastrointestinal cancers. Hematol Oncol Clin North Am. (2024) 38:693–710. doi: 10.1016/j.hoc.2024.01.007 38431494

[8] Sami SS Subramanian V Butt WM Bejkar G Coleman J Mannath J. High definition versus standard definition white light endoscopy for detecting dysplasia in patients with Barrett’s esophagus: HD endoscopy in Barrett’s surveillance. Dis Esophagus. (2015) 28:742–9. doi: 10.1111/dote.12283 25209721

[9] Chadebecq F Lovat LB Stoyanov D. Artificial intelligence and automation in endoscopy and surgery. Nat Rev Gastroenterol Hepatology. (2023) 20:171–82. doi: 10.1038/s41575-022-00701-y 36352158

[10] Feng X Zhu JH Hua ZL Zhou Q Shi AW Song TQ. Satisfaction in population-based cancer screening in a Chinese rural high-risk population: the Yangzhong early diagnosis and treatment of upper gastrointestinal cancer. BMC Health Serv Res. (2022) 22:675. doi: 10.1186/s12913-022-08076-1 35590328 PMC9121570

[11] Feng X Zhu JH Hua ZL Xu X Li Y Li J. Satisfaction and its determinants of rural upper gastrointestinal cancer screening in China: a preliminary cross-sectional study. BMJ Open. (2022) 12:e061483. doi: 10.1136/bmjopen-2022-061483 PMC9442482 36329609

[12] Xin Y Ren XH. Determinants of province-based health service utilization according to Andersen’ s Behavioral Model: a population-based spatial panel modeling study. BMC Public Health. (2023) 23:985. doi: 10.1186/s12889-023-15885-4 37237347 PMC10224305

[13] Song Y Liu J Yan S Ma M Tarimo CS Chen Y. Factors influencing health service utilization among 19,869 China’s migrant population: an empirical study based on the Andersen behavioral model. Front Public Health. (2025) 13:1456839. doi: 10.3389/fpubh.2025.1456839 39916717 PMC11798976

[14] Waddingham W Kamran U Kumar B Trudgill NJ Tsiamoulos ZP Banks M. Complications of diagnostic upper Gastrointestinal endoscopy: common and rare – recognition, assessment and management. BMJ Open Gastroenterology. (2022) 9:e000688. doi: 10.1136/bmjgast-2021-000688 PMC9806027 36572454

[15] Helgestad ADL Andersen B Njor SH Larsen MB. The association of demographic and socioeconomic variables with cancer screening participation: A national cross-sectional study of three cancer screening programs in Denmark. Heliyon. (2024) 10:e31163. doi: 10.1016/j.heliyon.2024.e31163 39044972 PMC11263647

[16] Ganta N Aknouk M Alnabwani D Nikiforov I Bommu VJL Patel V. Disparities in colonoscopy utilization for lower gastrointestinal bleeding in rural vs urban settings in the United States. World J Gastrointest Endosc. (2022) 14:474–86. doi: 10.4253/wjge.v14.i8.474 PMC9453311 36158630

[17] Greiner KA Engelman KK Hall MA Ellerbeck EF. Barriers to colorectal cancer screening in rural primary care. Prev Med. (2004) 38:269–75. doi: 10.1016/j.ypmed.2003.11.001 14766108

[18] Li J Xu M Liu T Zhang C. Regional differences, dynamic evolution and convergence of public health level in China. Healthcare. (2023) 11:1459. doi: 10.3390/healthcare11101459 37239745 PMC10218456

[19] Wolfe MK McDonald NC Holmes GM. Transportation barriers to health care in the United States: findings from the national health interview survey, 1997-2017. Am J Public Health. (2020) 110:815–22. doi: 10.2105/ajph.2020.305579 PMC7204444 32298170

[20] Dong EH Sun XT Xu T Zhang SX Wang T Zhang LF. Measuring the inequalities in healthcare resource in facility and workforce: A longitudinal study in China. Front Public Health. (2023) 11:1074417. doi: 10.3389/fpubh.2023.1074417 37006575 PMC10060654

[21] Qiu LJ Yang LS Li HR Wang L. The impact of health resource enhancement and its spatiotemporal relationship with population health. Front Public Health. (2023) 10:1043184. doi: 10.3389/fpubh.2022.1043184 36699901 PMC9868711

[22] Yang L Zhou Q Wang CZ Zhang DM Yuan T Li XP. Classification of health needs: a cluster analysis of older adults in urban areas. BMC Geriatr. (2023) 23:638. doi: 10.1186/s12877-023-04333-y 37814238 PMC10563358

[23] Alter BJ Anderson NP Gillman AG Yin Q Jeong J-H Wasan AD. Hierarchical clustering by patient-reported pain distribution alone identifies distinct chronic pain subgroups differing by pain intensity, quality, and clinical outcomes. PloS One. (2021) 16:e0254862. doi: 10.1371/journal.pone.0254862 34347793 PMC8336800

[24] Kavelaars X Mulder J Kaptein M. Bayesian multilevel multivariate logistic regression for superiority decision-making under observable treatment heterogeneity. BMC Med Res Methodol. (2023) 23:220. doi: 10.1186/s12874-023-02034-z 37798704 PMC10552398

[25] Jadallah K Khatatbeh M Mazahreh T Sweidan A Ghareeb R Tawalbeh A. Colorectal cancer screening barriers and facilitators among Jordanians: A cross-sectional study. Prev Med Rep. (2023) 32:102149. doi: 10.1016/j.pmedr.2023.102149 36852311 PMC9958352

[26] Bauer C Zhang KH Xiao Q Lu JC Hong YR Suk R. County-level social vulnerability and breast, cervical, and colorectal cancer screening rates in the US, 2018. JAMA Network Open. (2022) 5:e2233429. doi: 10.1001/jamanetworkopen.2022.33429 36166230 PMC9516325

[27] Mwachiro M Topazian HM Kayamba V Mulima G Ogutu E Erkie M. Gastrointestinal endoscopy capacity in Eastern Africa. Endosc Int Open. (2021) 9:E1827–e36. doi: 10.1055/a-1551-3343 PMC8589549 34790551

[28] Zia N Alam L Ashraf N. Endoscopic finding in patients presenting with lower gastrointestinal bleed-A study from A developing country. Pakistan Armed Forces Med J. (2021) 1):215. doi: 10.51253/pafmj.v71i1.6366

